# Expression of Colorectal Cancer Antigenic Protein Fused to IgM Fc in Chinese Cabbage (*Brassica rapa*)

**DOI:** 10.3390/plants9111466

**Published:** 2020-10-30

**Authors:** Ye-Rin Lee, Chae-Yeon Lim, Sohee Lim, Se Ra Park, Jong-Pil Hong, Jinhee Kim, Hye-Eun Lee, Kisung Ko, Do-Sun Kim

**Affiliations:** 1 Vegetable Research Division, National Institute of Horticultural and Herbal Science, Rural Development Administration, Wanju-gun 55365, Korea; lyr1219@korea.kr (Y.-R.L.); chaeyeon0101@naver.com (C.-Y.L.); hjp3467@daum.net (J.-P.H.); sayzinni@korea.kr (J.K.); helee72@korea.kr (H.-E.L.); 2 Department of Medicine, College of Medicine, Chung-Ang University, Seoul 06974, Korea; annysh520@gmail.com (S.L.); stojr1988@naver.com (S.R.P.)

**Keywords:** *Agrobacterium*, colorectal carcinoma, EpCAM, plant molecular biopharming, recombinant vaccine, transgenic chinese cabbage

## Abstract

The epithelial cell adhesion molecule (EpCAM) is a tumor-associated antigen and a potential target for tumor vaccine. The EpCAM is a cell-surface glycoprotein highly expressed in colorectal carcinomas. The objective of the present study is to develop an edible vaccine system through *Agrobacterium*-mediated transformation in Chinese cabbage (*Brassica rapa*). For the transformation, two plant expression vectors containing genes encoding for the EpCAM recombinant protein along with the fragment crystallizable (Fc) region of immunoglobulin M (IgM) and Joining (J)-chain tagged with the KDEL endoplasmic reticulum retention motif (J-chain K) were constructed. The vectors were successfully transformed and expressed in the Chinese cabbage individually using *Agrobacterium*. The transgenic Chinese cabbages were screened using genomic polymerase chain reaction (PCR) in T_0_ transgenic plant lines generated from both transformants. Similarly, the immunoblot analysis revealed the expression of recombinant proteins in the transformants. Further, the T_1_ transgenic plants were generated by selfing the transgenic plants (T_0_) carrying EpCAM–IgM Fc and J-chain K proteins, respectively. Subsequently, the T_1_ plants generated from EpCAM–IgM Fc and J-chain K transformants were crossed to generate F_1_ plants carrying both transgenes. The presence of both transgenes was validated using PCR in the F_1_ plants. In addition, the expression of Chinese cabbage-derived EpCAM–IgM Fc × J-chain K was evaluated using immunoblot and ELISA analyses in the F_1_ plants. The outcomes of the present study can be utilized for the development of a potential anti-cancer vaccine candidate using Chinese cabbage.

## 1. Introduction

Plant expression systems, such as those in tomatoes [1,2], carrots [3], bananas [4], spinach [5], lettuce [6], and tobacco [7,8] have been established to produce valuable recombinant proteins, including therapeutic enzymes, vaccines, and antibodies. Among horticultural crops, Chinese cabbage has been recently recognized as a potential candidate to produce such valuable recombinant proteins, because it has a reasonable total soluble protein capacity relative to plant biomass [9]. In general, a large amount of soluble protein in plant biomass can provide better production of recombinant proteins, thereby enhancing the possibility of an efficient plant-based bioreactor system [10]. Chinese cabbage transformation protocols are well established for the overexpression of transgenes, which can impart resistance to plant pathogenesis [11,12,13] and tolerance to harsh environments, such as drought and salinity [14,15,16], and it can also improve physiological traits, such as tolerance to calcium deficiency [17]. There are several advantages of using Chinese cabbage for the production of therapeutic proteins. Chinese cabbage leaf biomass can be directly used in oral application without the purification of recombinant vaccines. Isolates of the targeted recombinant proteins can be easily purified from its leaf biomass. Additionally, Chinese cabbage is easy to manipulate by breeding and establishing mass seed production with self-compatibility or self-incompatibility [18]. However, the application of Chinese cabbage for therapeutic protein production has not yet been actively studied.

Among the valuable recombinant proteins expressed in plants, an epithelial cell adhesion molecule (EpCAM) is a tumor-associated antigen (TAA) that has been shown to be highly expressed on cancer cells over the last several decades and expressed using diverse heterologous expression including plants such as Swiss chard (*Beta vulgaris* subsp. Vulgaris), *Nicotiana tabaccum*, and *N. Benthamiana* [19,20,21,22,23,24,25,26]. Plants have glutaminyl cyclases to catalyze the formation of pyroglutamic acid at the N-terminus of proteins, which is important for its immunogenicity [27,28]. Thus, it is assumed that N-terminal glutamine could be modified to pyroglutamate in diverse transgenic plants species, including Chinese cabbage. The EpCAM cancer antigenic protein can be used to prevent and inhibit cancer; hence, it is a vaccine candidate [23]. The EpCAM protein is often fused to the fragment crystallizable (Fc) region of immunoglobulin G (IgG Fc), having the advantages of Fc fusion, including enhanced protein stability, ease of application for affinity chromatography, and enhanced biofunctions [24,29,30]. Therefore, the Fc-fusion strategy has also been applied to the EpCAM-Fc fusion cancer vaccine expressed in insect and plant systems [31,32]. The plant-derived EpCAM fused to IgG Fc induced a humoral immune response in BALB/c mice [29,30]. In previous studies, the Fc fused to EpCAM originated from IgG, eventually forming IgG monomeric structures [33,34]. Recently, the fusion of Fc originating from IgA and IgM, for therapeutic recombinant proteins, has been applied to form dimeric or polymeric structures that have improved biofunctions [30,31]. In particular, IgM-like protein structures can provide hexameric or pentameric structures that have advantages, such as an ease of recognition of dendritic cells, thereby efficiently presenting an antigen for an induction of the immune response [35]. The co-expression of both IgM and Joining (J)-chain could avoid the hexameric structures that cause cytosolic responses through IgM-C_1_q complement-dependent cytotoxicity [36]. Indeed, such an antibody-like protein complex could have beneficial biological and chemical properties as a vaccine by targeting the antigen-presenting cells (APCs), protein–A/G-derived affinity purification, and protein in vivo stability [37]. Additionally, the Fc-fusion strategy could provide higher expression levels and stability in heterologous expression systems, eventually leading to better yield in a plant expression system [29,31,33].

**Figure 1:**
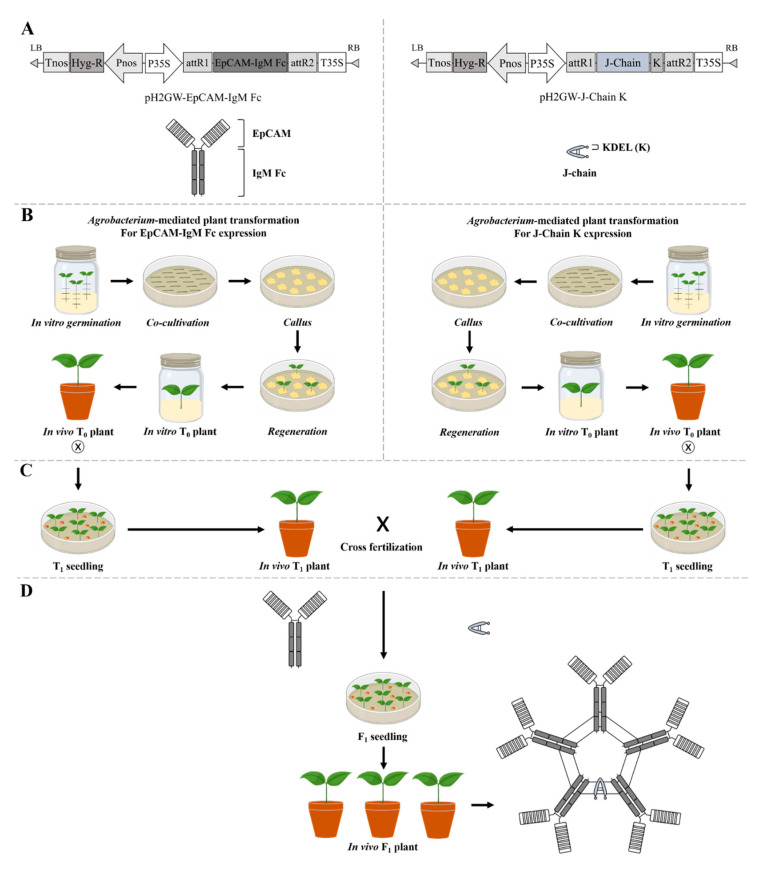
Schematic diagram for *Agrobacterium*-mediated Chinese cabbage transformation and generation of transgenic plants carrying both epithelial cell adhesion molecule–immunoglobulin M fragment crystallizable (EpCAM–IgM Fc) and Joining (J)-chain K. (**A**) Plant expression vectors to express EpCAM–IgM Fc and J-chain K for *Agrobacterium*-mediated transformation. The expected quaternary structure of EpCAM–IgM Fc in T_0_ transgenic plants carrying the transgene encoding EpCAM–IgM Fc is hexameric. The expected protein structure of the J-chain K in T_0_ transgenic plants carrying the transgene encoding J-chain K is hook-like. (**B**) Generation of T_0_ transgenic plants carrying EpCAM–IgM Fc and J-chain K obtained from *Agrobacterium*-mediated transformation. (**C**) T_1_ transgenic plants with EpCAM–IgM Fc and J-chain K obtained from self-crossing of T_0_ transgenic plants with EpCAM–IgM Fc and J-chain K. Cross-fertilization between T_1_ transgenic plants carrying EpCAM–IgM Fc and J-chain K transgenes after selfing of T_0_ plant generation. (**D**) F_1_ transgenic plants carrying both transgenes encoding EpCAM–IgM Fc and J-chain K obtained from crossing between T_1_ transgenic plants carrying transgenes encoding EpCAM–IgM Fc and T_1_ transgenic plants carrying transgenes encoding J-chain K. The expected quaternary structure of EpCAM–IgM Fc × J-chain K in transgenic plant F_1_ is pentameric.

In the present study, the Fc of IgM was fused to EpCAM to generate polymeric protein structures as a cancer vaccine in the Chinese cabbage expression system. Additionally, the J-chain was expressed in Chinese cabbage. Both transgenic plants carrying EpCAM fused to IgM Fc, and J-chain recombinant protein transgenes were crossed to generate F_1_ transgenic plants and investigate the potential of Chinese cabbage as a plant bioreactor to produce highly valuable antigen–Fc complex proteins for cancer immunotherapy (Figure 1).

## 2. Results

### 2.1. Agrobacterium-Mediated Transformation and Regeneration of T_0_ Transgenic Chinese Cabbage

**Figure 2:**
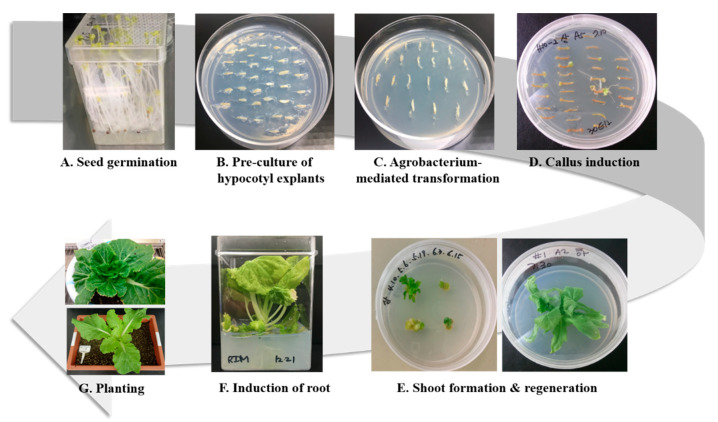
*Agrobacterium*-mediated Chinese cabbage transformation and generation of transgenic Chinese cabbage carrying transgenes encoding EpCAM–IgM Fc and J-chain K. (**A**) In Vitro seed germination to generate Chinese cabbage seedlings. (**B**) In Vitro preculture of hypocotyls in media. (**C**) *Agrobacterium*-inoculated hypocotyls incubated in in vitro regeneration media. (**D**) Callus generated from in vitro callus-inducing media. (**E**) Shoot regeneration from calli under the regeneration media. The left shows newly regenerated shoots from the callus. The right shows shoots after further growth following transfer to new regeneration media. (**F**) Root induction from the regenerants in root-inducing media. (**G**) In Vivo T_0_ transgenic Chinese cabbage growth after transplanting into pots with soil from the in vitro root-induced transgenic plants.

*Agrobacterium*-mediated transformation was conducted to transfer genes encoding EpCAM–IgM Fc or J-chain K to Chinese cabbage (Figure 2). Three hundred hypocotyl pieces of Chinese cabbage were applied to transform plant expression vectors (pH2GW EpCAM–IgM Fc, and J-chain K) (Figure 1A,B). After co-cultivation with *Agrobacterium*, the hypocotyl pieces were transferred onto regeneration media (Figure 2C,D). A callus began to form at the cut ends of 30–40% of the hypocotyls within 2 weeks (Figure 2D). Adventitious shoots were observed from calli after 3–4 weeks (Figure 2D,E). The shoots were isolated from the explants and transferred onto the regeneration medium with 6 mg/L phosphinotricin (PPT) when they were at least 4–5 mm in length (Figure 2E,F). Most shoots showed vigorous regenerative growth on the new selective medium. However, some of the shoots became etiolated and died one week later. These results suggested that there were escapees from the selective medium culture. In some cases, the leaves of regenerated plants exhibited chlorosis, indicating that these plants might be chimeras.

### 2.2. Presence of Transgenes Encoding EpCAM–IgM Fc and J-Chain K in T_0_ Transgenic Plants

**Figure 3:**
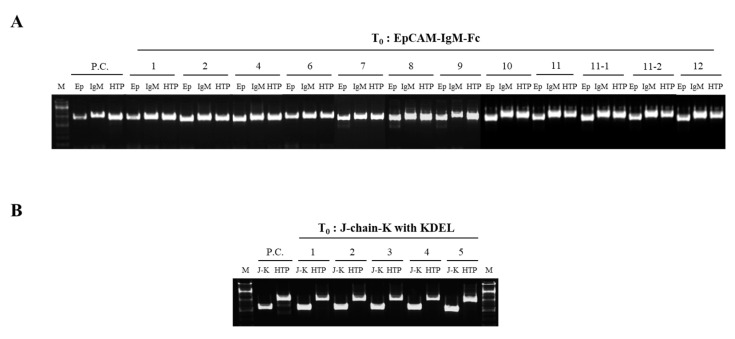
A polymerase chain reaction (PCR) analysis to confirm the transgenes encoding EpCAM–IgM Fc and J-chain K in T_0_ transgenic lines. (**A**) Transgenic lines (#1–12) carrying the transgene encoding EpCAM–IgM Fc were tested by PCR using three different primer sets for EP, IgM Fc, and HTP. PC is a positive control; M, size marker; Ep, EpCAM (837 bp); IgM, Fc of IgM (1053 bp); HTP, hygromycin phosphotransferase (1023 bp); non-transgenic plant (NT). (**B**) Transgenic lines (#1–5) carrying a transgene encoding J-chain K. J-K, J-chain fused to KDEL endoplasmic reticulum retention signal; J-K, J-chain K (543 bp); hygromycin phosphotransferase (HTP) (1023 bp); non-transgenic plant (NT).

A polymerase chain reaction (PCR) analysis of the genomic DNA was conducted to confirm the presence of transgenes in transgenic plants (EpCAM–IgM Fc) and (J-chain K) randomly selected Chinese cabbage regenerants obtained by transforming each vector (Figure 3A,B, respectively). All tested regenerants obtained from transformations using plant expression vectors carrying the gene encoding EpCAM–IgM Fc had amplified bands at 837 bp (EpCAM), 1053 bp (IgM Fc), and 1023 bp (hygromycin phosphotransferase (HTP)) (Figure 3A). All tested regenerants obtained from transformation using the plant expression vector carrying the gene encoding the J-chain K had the expected amplified J-chain K band at 543 bp (Figure 3B).

### 2.3. Expression of EpCAM–IgM Fc in T_0_ Transgenic Plants

**Figure 4:**
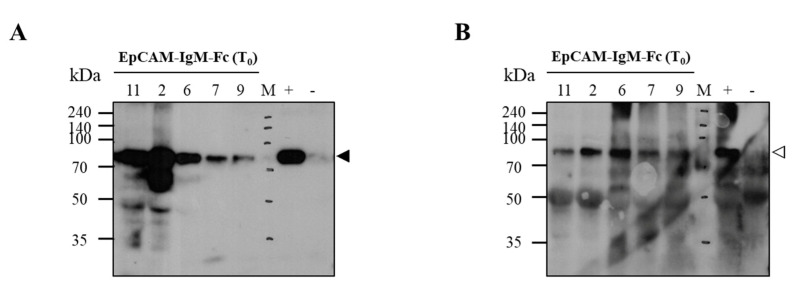
Immunoblot analysis to confirm the expression of EpCAM–IgM Fc in T_0_ transgenic lines. (**A**) T_0_ transgenic plants #2, 6, 7, 9, and 11 were tested by goat anti-IgM Fc µ-chain conjugated to HRP. M, protein marker; +, tobacco plant EpCAM–IgM Fc as a positive control; −, non-transgenic plant as a negative control. (**B**) T_0_ transgenic plants #2, 6, 7, 9, and 11 were tested by mouse anti-human EpCAM as a primary first antibody and goat anti-mouse IgG 2a heavy chain conjugated to HRP as a secondary antibody. M, protein marker; +, tobacco plant EpCAM–IgM Fc as a positive control (50 ng); −, non-transgenic plant as a negative control; hygromycin phosphotransferase (HTP).

Expression of the EpCAM–IgM Fc protein in leaf tissues of transgenic plants was analyzed using Western blotting (Figure 4). In the IgM Fc detection immunoblot, all randomly selected transgenic lines (2,6,7,9,11) showed an approximately 75 kDa protein band similar to that of the positive control (Figure 4A black arrowhead). In the EpCAM detection immunoblot, all lines (2,6,7,9,11) of transgenic plants showed the expected bands for EpCAM–IgM Fc (Figure 4A) with an EpCAM–IgM Fc-sized protein band (Figure 4B white arrowhead). No band was observed in the non-transgenic plants (Figure 4).

### 2.4. Presence of Transgenes Encoding EpCAM–IgM Fc and J-Chain K in T_1_ Transgenic Plants

**Figure 5:**
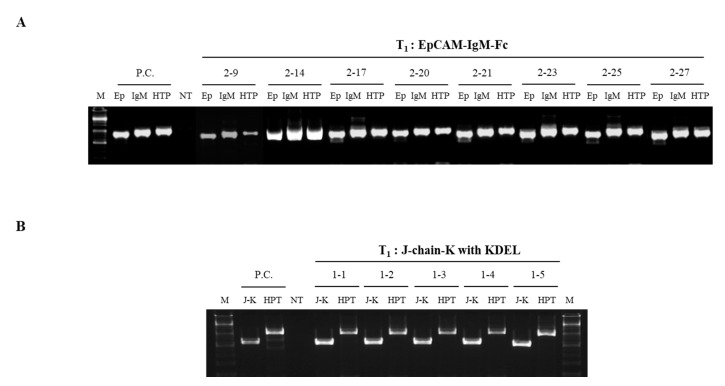
A polymerase chain reaction (PCR) analysis to confirm the transgenes encoding EpCAM–IgM Fc and J-chain K in the T_1_ transgenic lines. (**A**) T_1_ transgenic lines (#2–9, 2–14, 2–17, 2–20, 2–21, 2–23, 2–25, and 2–27) carrying the transgene encoding EpCAM–IgM Fc were tested by PCR using three different primer sets for EP, IgM Fc, and HTP. PC is a positive control; M, size marker; Ep, EpCAM (837 bp); IgM, Fc of IgM (1053 bp); HTP, hygromycine phosphotransferase (1023 bp); non-transgenic plant (NT). (**B**) T_1_ transgenic lines (#1–1, 1–2, 1–3, 1–4, and 1–5) carrying the transgene encoding J-chain K. J-K, J-chain fused to KDEL endoplasmic reticulum retention signal; J-K, J-chain K (543 bp); hygromycin phosphotransferase (HTP) (1023 bp); non-transgenic plant (NT).

The T_0_ transgenic line #2, with the highest expression of EpCAM–IgM Fc, was self-crossed to generate a T_1_ line #2 (Figure 1; Figure 4). PCR was performed for the presence of transgenes in eight (EpCAM–IgM Fc) T_1_ plants (Figure 5). All tested T_1_ plants obtained from the transformation using the plant expression vector carrying the gene encoding EpCAM–IgM Fc had the expected amplified bands at 837 bp (EpCAM), 1053 bp (IgM–Fc), and 1023bp (HTP) (Figure 5A). In the transgenic plant carrying the J-chain K transgene, the transgenic line 1 was self-crossed to generate T_1_. Transgenic plants in T_1_ carrying the J-chain K transgene were randomly selected for PCR testing (Figure 5B). All of the tested T_1_ plants obtained from selfing had the expected amplified band at 543 bp (J-chain K) (Figure 5B).

### 2.5. Expression of EpCAM–IgM Fc in T_1_ Transgenic Plants

**Figure 6:**
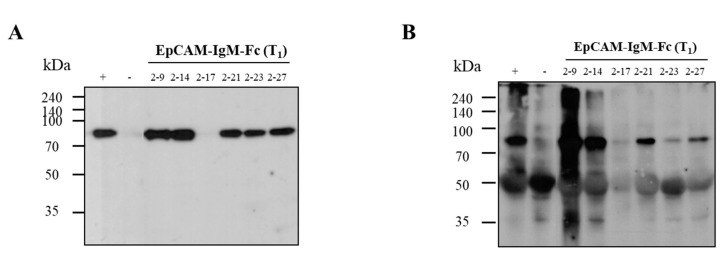
Immunoblot analysis to confirm the expression of EpCAM–IgM Fc in representative T1 transgenic lines. (**A**) T1 transgenic plants #2–9, 2–14, 2–17, 2–21, 2–23, and 2–27 were tested by goat anti-human IgM Fc µ-chain conjugated to HRP. M, protein marker; +, tobacco plant EpCAM–IgM Fc as a positive control; −, non-transgenic plant as a negative control. (**B**) T1 transgenic plants #2–9, 2–14, 2–17, 2–21, 2–23, and 2–27 were tested by mouse anti-human EpCAM as the first antibody and goat anti-mouse IgG 2a conjugated to HRP as the second antibody. M, protein marker; +, tobacco plant EpCAM–IgM Fc as a positive control (50 ng); −, non-transgenic plant as a negative control; hygromycin phosphotransferase (HTP).

In all tested T_1_ transgenic lines, expression of the EpCAM–IgM Fc protein was confirmed by Western blotting (Figure 6). In the IgM Fc detection immunoblot, all tested transgenic lines (#2–9, 2–14, 2–28, 2–35, and 2–54), except for lines 2–17, showed a strong protein band (75 kDa) (Figure 6A). In the EpCAM detection immunoblot, the #2–9, 2–14, and 2–21 lines showed the strongest protein band density, whereas the #2–17, 2–23, and 2–27 lines had weak protein bands (75 kDa) (Figure 6B). Additionally, 50 kDa size bands were also observed in all lines, including the non-transgenic control line. No band was observed in the non-transgenic plants (Figure 6). It is speculated that the 50 kDa protein bands are Rubisco proteins, which are often detected in plant total soluble proteins.

### 2.6. Presence of Transgenes Encoding EpCAM–IgM Fc and J-Chain K in F_1_ Transgenic Plants Obtained between EpCAM–IgM Fc and J-Chain K T_1_ Plants

**Figure 7:**
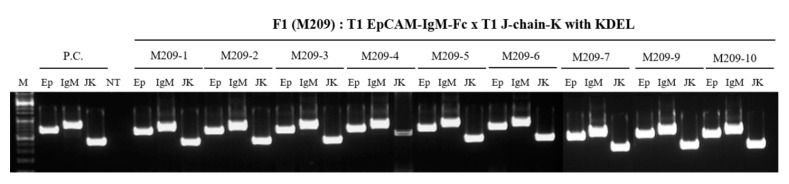
A polymerase chain reaction (PCR) analysis to confirm the transgenes encoding EpCAM–IgM Fc and J-chain K in F_1_ transgenic lines obtained by crossing between T_1_ EpCAM Fc transgenic plants (#2) and T_1_ J-chain transgenic lines (#1). F_1_ transgenic lines (#M209-1, M209-2, M209-3, M209-4, M209-5, M209-6, M209-7, M209-9, and M209-10) carrying a transgene encoding EpCAM–IgM Fc were tested by PCR using three different primer sets for Ep, IgM Fc, and HTP. positive control (PC); size marker (M); Ep, EpCAM (837 bp); IgM, Fc of IgM (1053 bp); J-K, J-chain K (543 bp); non-transgenic plant (NT); hygromycine phosphotransferase (HTP).

The F_1_ plants were obtained from crossing EpCAM–IgM Fc and J-chain K T_1_ plants. The presence of transgenes encoding EpCAM–IgM Fc and J-chain K was confirmed in the F_1_ plants using PCR. In randomly selected F_1_ plants, all tested F_1_ lines showed the presence of the transgenes EpCAM (837 bp), IgM Fc (1053 bp), and J-chain K (543 bp), except for M209-8, in which only the J-chain K gene was detected without the EpCAM and IgM Fc genes (Figure 7).

### 2.7. Expression of EpCAM–IgM Fc in F_1_ Transgenic Plants Obtained by Crossing EpCAM–IgM Fc and J-Chain K T_1_ Plants

**Figure 8:**
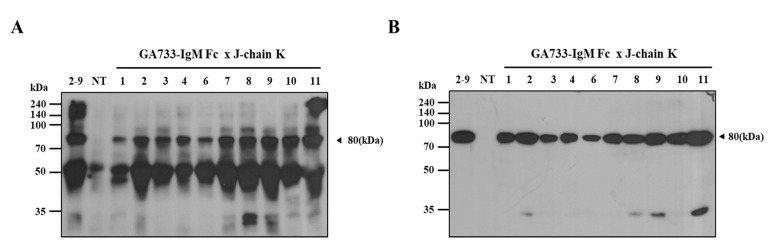
Immunoblot analysis to confirm the expression of EpCAM–IgM Fc in representative F_1_ transgenic lines. (**A**) F_1_ transgenic plants M209-1–209-10 were tested by goat anti-IgM Fc µ-chain conjugated to HRP. protein marker (M); +, tobacco plant EpCAM–IgM Fc as a positive control; − non-transgenic plant as a negative control. (**B**) F_1_ transgenic plants M209-1–209-10 were tested by mouse anti-human EpCAM as the first antibody and goat anti-mouse IgG 2a conjugated to HRP as the second antibody. 2–9, T_1_ transgenic plant #2–9 (Figure 6); +, tobacco plant EpCAM–IgM Fc as a positive control (50 ng); −, non-transgenic plant (NT) as a negative control; hygromycin phosphotransferase (HTP).

In the F_1_ transgenic lines with positive PCR results for EpCAM, IgM Fc, and J-chain K transgenes, the expression of EpCAM–IgM Fc was confirmed by Western blotting (Figure 8A). In the IgM Fc detection immunoblot, all tested F_1_ transgenic lines (#1–9) showed the 75 kDa size protein band. Furthermore, in the EpCAM detection immunoblot, all tested F_1_ transgenic lines (#1–9) showed the 75 kDa size protein band (Figure 8B). No bands were observed in the non-transgenic plants (Figure 8).

### 2.8. Binding Affinity of the Anti-IgM Fc µ-Chain Antibody to Chinese Cabbage-Derived EpCAM-IgM Fc × J-Chain K (EpCAM-IgM FcC × J-Chain KC).

**Figure 9:**
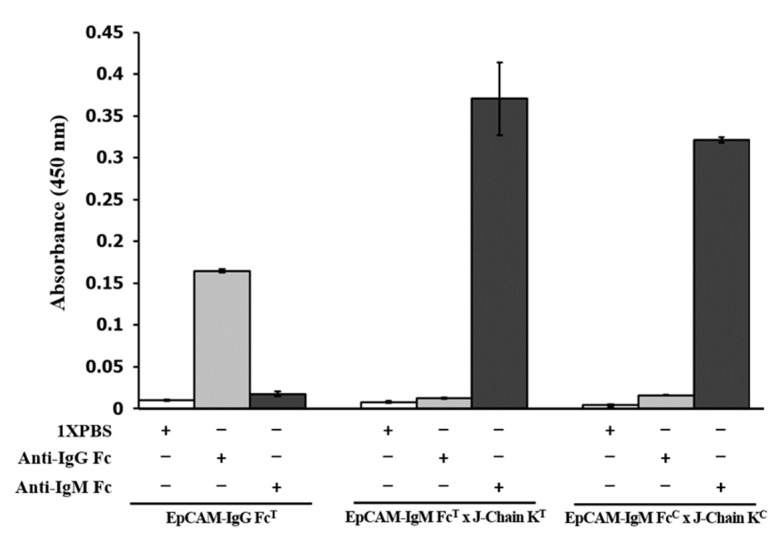
Enzyme-linked immunosorbent assay (ELISA) was performed to confirm binding interaction between anti-IgM Fc µ-chain antibody and EpCAM–IgM Fc × J-ChainK purified from F_1_ transgenic Chinese cabbage plant. Each ELISA plate well was coated with 10 ng of the purified Chinse cabbage-derived EpCAM–IgM Fc × J-ChainK (EpCAM–IgM Fc^C^ × J-ChainK^C^), tobacco-derived EpCAM–IgM Fc × J-ChainK (EpCAM–IgM Fc^T^ x J-ChainK^T^), or tobacco-derived EpCAM–IgG Fc (EpCAM–IgG Fc ^T^). Each anti-IgM Fc µ-chain antibody and anti-IgG Fc antibody conjugated to HRP was applied to the coated well. Error bars indicate standard deviation (* *p* < 0.05); hygromycin phosphotransferase (HTP).

Enzyme-linked immunosorbent assay (ELISA) was conducted for the recognition of Chinese cabbage-derived EpCAM–IgM Fc^C^ × J-chain KC using an anti-IgM Fc µ-chain antibody (Figure 9). In the anti-IgM Fc µ-chain antibody treatment group, both EpCAM–IgM Fc^T^ × J-ChainK^T^ and EpCAM–IgM Fc^C^ × J-ChainK^C^ showed high absorbance levels (0.367 and 0.315, respectively), whereas EpCAM–IgG Fc^T^ had almost no absorbance signal (0.02) (Figure 9). However, in the anti-IgG Fc treatment group, both EpCAM–IgM Fc^T^ × J-ChainK^T^ and EpCAM–IgM Fc^C^ × J-ChainK^C^ showed almost no absorbance signal (0.01 and 0.018, respectively), whereas EpCAM–IgG Fc^T^ showed an absorbance signal (0.165) (Figure 9). In the 1X PBS treatment group, all three samples lacked an absorbance signal (Figure 9).

## 3. Discussion

This study demonstrated the successful expression of EpCAM–IgM Fc as anti-colorectal cancer IgM Fc fusion antigenic proteins, creating a candidate for a cancer vaccine in transgenic T_0_ and T_1_ Chinese cabbage, including F_1_ plants from a crossing between EpCAM–IgM Fc and J-chain K T_1_ transgenic plants. Transgenic Chinese cabbage expressing EpCAM–IgM Fc and J-chain K were obtained from *Agrobacterium*-mediated transformation. PCR analysis revealed that all tested T_0_ transgenic plants carrying EpCAM–IgM Fc and J-chain K transgenes had PCR bands of both transgenes, indicating that these genes were properly embedded in the plant genome. Western blotting showed variable expression of the EpCAM–IgM Fc transgene in the T_0_ transgenic plants. In EpCAM–IgM Fc, among the tested T_0_ transgenic plants, transgenic line #2 had the highest level of EpCAM–IgM Fc. Thus, EpCAM–IgM Fc transgenic line #2 was selected for self-crossing to generate T_1_ plants. Regarding the J-chain K, according to Western blot analysis, its expression was not observed in the T_0_ transgenic plants.

In both EpCAM–IgM Fc and J-chain K T_1_ transgenic plants, the existence of EpCAM–IgM Fc and J-chain K transgenes was confirmed by PCR, respectively. However, Western blotting revealed that only the EpCAM–IgM Fc protein expression was detected in the T_1_ plants, and the J-chain K protein was not detected. It appeared that J-chain protein expression is difficult to detect. Currently, there are no reports demonstrating J-chain expression in transgenic plants. However, the function of the J-chain is active when it is co-expressed with IgM or immunoglobulin A (IgA) antibodies [36]. Despite a barely detectable J-chain protein level, it has vital activity when it assembles with IgM Fc or IgA Fc to generate pentameric or dimeric structures, respectively [38].

The T_1_ transgenic line highly expressing EpCAM–IgM Fc proteins was selected to cross with the T_1_ transgenic line carrying the J-chain K transgene to generate F_1_ plants carrying two transgenes. Since transgenic Chinese cabbage plants have the ability of self-fertilization and cross-fertilization, this crop has been efficiently bred to build useful characteristics in terms of growth physiology, taste, and disease resistance [39,40,41]. In this study, we used self- and cross-fertilization of Chinese cabbage to obtain the F_1_ plants having two different transgenes encoding EpCAM–IgM Fc and J-chain K. The immunoglobulin Fc-fusion protein is the protein linked to the immunoglobulin Fc fragment. The Fc-fused proteins obtain beneficial biological and pharmacological effects from the Fc fragment [42,43]. The Fc fragment remarkably enhances the plasma half-life of their fused proteins or peptides through its FcRn interaction, eventually prolonging their therapeutic activities [43]. Additionally, the Fc fragment interacts with Fc receptors on immune cells to provoke antigen presentation, inducing immune responses [44,45]. Furthermore, regarding protein expression, the Fc-fused proteins in general exhibit better protein expression [29]. The Fc-fused protein can be purified by Protein-A affinity chromatography [42]. In general, there are three major Ig Fc fragment forms, IgG, IgA, and IgM. The IgG and IgA-Fc fused proteins can be assembled in monomeric and dimeric forms, respectively [46]. The IgM Fc can be assembled to polymerize through disulfide bonding localized to the junction portions of CH2 and CH3 of the Fc fragment [35]. The J-chain has important functions in the assembly with IgA and IgM, acting as a glue between the Fc regions of each antibody [35]. In IgA, the J-chain enhances the dimerization of IgA and its secretion process [47]. In IgM, J-chain forms the pentameric structure from the hexameric structure [35]. The pentameric structures are less effective at activating complements than the hexameric structures, reducing damage to epithelial membranes. The J-chain on IgM might sterically hinder the binding of complement component 1q (C1q) and thereby decrease the cytolytic activity of pentameric IgM [48]. Therefore, the J-chain should be co-expressed with the IgM–Fc fusion anti-colorectal cancer vaccine candidate. The two expression cassettes can be combined and transferred into one *Agrobacterium*-mediated transformation. However, in this study, each transgene was individually transferred to each Chinese cabbage plant, and these plants were successfully cross-fertilized to obtain both transgenes. To confirm whether an anti-human IgM Fc µ-chain antibody can recognize Chinese cabbage-derived EpCAM–IgM Fc × J-chain K, an ELISA analysis was conducted. The binding reaction between EpCAM–IgM Fc × J-chain K and the anti-human IgM Fc µ-chain antibody showed an absorbance signal indicating that the EpCAM–IgM Fc × J-chain K was structurally formed to be recognized by the anti-IgM Fc µ-chain antibody. In previous studies, EpCAM alone and fused with IgG Fc have been expressed in diverse plants [26,27,28], however, the IgM–Fc fused EpCAM is the first report in this study. In this current study, a glycosylation study with the EpCAM–IgM × J-chain K was not conducted, which affects its immunogencitiy. It has been reported that the *Brasscia* species has glycosylation apparatus for protein glycosylation [49,50]. In the current study, it is speculated that the EpCAM–IgM Fc assembled with J-chain K, retaining it inside endoplasmic reticulum (ER), harboring mainly an oligomannose type. Thus, in the future, glycosylation should be further investigated.

All considered, we confirmed that each EpCAM–IgM Fc and J-chain K transgene expression cassette was successfully transferred to the Chinese cabbage, and cross-fertilization between both transgenic plants carrying one of the transgenes generated F_1_ transgenic seedlings carrying both transgenes. These results revealed that both transgenes were stably inserted, and the EpCAM–IgM Fc protein gene was expressed in F_1_ transgenic plants reproduced from crossing between EpCAM–IgM Fc and J-chain K T_1_ transgenic plants. Furthermore, these results suggested that two genes can co-exist through cross-breeding in Chinese cabbage T_1_ transgenic plants. In conclusion, the transgenic Chinese cabbage expressing EpCAM–IgM Fc can be applied to express anti-colorectal cancer IgM Fc fusion recombinant vaccine candidate proteins.

## 4. Materials and Methods

### 4.1. Plant Expression Vector and Agrobacterium Strain

The gene encoding the human EpCAM protein (Thr17-Lys265, GenBank accession no. BC014785) was fused to the gene encoding human IgM Fc polypeptides (Leu103-Tyr453, GenBank accession no. X57086) to construct the EpCAM–IgM Fc fusion protein, and this was cloned under the CaMV promoter in the plant expression vector pRCV2 to generate pRCV2 EpCAM–IgM Fc carrying the hygromycin phosphotransferase (*hpt*) gene for Chinese cabbage *(B. rapa* L. ssp. *pekinensis*) transformation [45]. The 30 amino acids (MATQRRANPSSLHLITVFSLLAAVVSAEVD) as an ER signal peptide from *Nicotiana plumbaginifolia* was fused to N-terminus of the cleavage site (Thr17-Ala23). Chinese cabbage was transformed with the plant expression vector pRCV2 EpCAM–IgM Fc (Figure 1A). This vector was constructed by ligating pCAMBIA 1301 with pBluescript II KS (+) (Stratagene, San Diego, CA, USA) according to a previous study [12]. The *Agrobacterium tumefaciens* strain LBA 4404 carrying pRCV2 EpCAM–IgM Fc was applied for plant transformation. Agrobacteria were grown in a yeast extract peptone (YEP) medium.

### 4.2. Plant Material and Preparation

Chinese cabbage Seoul (Dong-bu Seed, Seoul, Korea) was used for *Agrobacterium*-mediated transformation to express EpCAM–IgM Fc and the J-chain tagged with the KDEL endoplasmic reticulum retention motif (J-chain K) (Figure 1B). The seeds were submerged in 70% ethanol for 1 min. Then, the seeds were vigorously shaken in 30% commercial Clorox (The Clorox company, Oakland, CA, USA) (1.6% hypochlorite) plus 0.1% Tween-20 (USB, Cleveland, OH, USA) for 20 min. Then, they were rinsed three times with water. The seeds were germinated on an MS medium [51] and cultivated in vitro to grow hypocotyls 4–5 cm long for 7 days under a 16 h photoperiod. Following this, the hypocotyls were dissected, avoiding the shoot apex, and quickly cut into 7–8 mm segments for *Agrobacterium*-mediated transformation.

### 4.3. Transformation and Selection Procedures

One mL of *Agrobacterium* cell stock was cultured in 50 mL of a YEP medium containing kanamycin (50 mg/L) and acetosyringone (5 mg/L) until an optical density (OD) 600 value of 1.0 was obtained. The *Agrobacterium* cells were pelleted and washed. Then, they were resuspended in 50 mL of the YEP medium. *Agrobacterium* cells were applied to infect hypocotyl explants by immersing them in the bacterial inoculum for 10 min (Figure 1B). The hypocotyl explants were blotted on sterile filter paper and placed on a co-cultivation medium containing acetosyringone (5 mg/L) at pH 5.2–5.7. After 3 days of co-cultivation, the explants were washed with an MS liquid medium supplemented with cefotaxime (200 mg/L) and transferred to an MS selection medium containing 3% sucrose, IBA (4 mg/L), NAA (3 mg/L), AgNO_3_ (4 mg/L), acetosyringone (5 mg/L), cefotaxime (200 mg/L), hygromycin (10 mg/L), and 0.8% plant agar (pH 5.6). After cultivation for 3 and 8 weeks, the number of calli and shoots that formed on the explants were determined, respectively (Figure 1B). Then, the regenerated shoots were transferred to a rooting medium that consisted of 1/2-strength MS medium, 3% sucrose, cefotaxime (200 mg/L), and 0.7% plant agar (pH 5.8).

### 4.4. PCR Amplification from Genomic DNA of Plant Leaf

To confirm the existence of transgene encoding EpCAM–IgM Fc, genomic DNA was prepared from the fresh leaves of transgenic and non-transgenic Chinese cabbage plants according to the DNA extraction kit (RBC Bioscience, Taipei, Taiwan) using the mini-prep method and protocol. DNA concentration was measured using a Nanovue^TM^ plus spectrophotometer (GE Healthcare, Boston, MA, USA) and adjusted to 20 ng/μL, and the DNA was used for PCR amplification. The status of transgenic plants was confirmed by PCR using HTP (hygromycine) and target gene (EpCAM, IgM, and J-Chain K) specific primers. The reaction solution for PCR analysis contained 20 ng of gDNA, 5.0 μL of Takara buffer mixture buffer (Takara, Kusatsu, Japan), 1.0 μL each of 10 pmol/μL of primer set, and autoclaved distilled water, to reach a total volume of 20 μL. The primer set for EpCAM (837 bp, forward primer (F)-GCA GGC TAT GGC TAC TCA ACG AAG G/reverse primer (R)-CTC GAG TTT TAG ACC CTG CAT TGA G), IgM (1053 bp, F-CTC GAG CTT CCA GTG ATT GCT GAG C/R-CTC GAG TCA GTA GCA GGT GCC AGC TGT G), J-chain K (543 bp, F-GCA GGC TAT GGC CAA GAA CCA TTT GC/R-AGT TCA TCT TTG TCA GGA TAG CAG G), and hygromycine (HPT, 1023 bp, F-CTA TTC CTT TGC CCT CGG ACG GC/R-ATG AAA AAG CCT GAA CTC ACC GCG ACG) genes were used for DNA amplification to confirm the presence of the transgene in the plants. The PCR reaction was performed as follows: denaturation at 95 ℃ for 10 min in the beginning, a repeated cycling step of denaturation at 95 ℃ for 1 min, annealing at 56 ℃ for 40 s, and extension at 72 ℃ for 1 min (35 times). This was followed by a final denaturation at 72 ℃ for 10 min. A non-transgenic Chinese cabbage plant was used as a negative control, whereas plant expression vectors containing the EpCAM–IgM Fc and J-chain K genes were used as positive controls.

### 4.5. Western Blot

To confirm the expression of EpCAM–IgM Fc in transgenic Chinese cabbage, 100 mg of leaf tissue was harvested from in vitro tissue culture and homogenized to generate leaf extracts in 1× PBS. A volume of 20 μL of leaf extract samples (100 mg of leaf/300 μL) mixed with 5 μL of protein loading buffer (1 M Tris-HCl, 50% glycerol, 10% SDS, 5% 2-mercaptoethanol, and 0.1% bromophenol blue) was loaded on 10% SDS-PAGE and transferred to a nitrocellulose membrane (Millipore, Billerica, MA, USA). The membrane was incubated in blocking buffer (5% skim milk (Fluka, Buchs, Switzerland) in Tris-Buffered Saline (TBS) plus 0.5% (*v/v*) Tween 20). The blot was incubated for 1 h 30 min at room temperature (RT) with either mouse anti-human EpCAM antibody # MAB960-500 (R&D Systems, Minneapolis, MN, USA) diluted in blocking buffer at 1:500 or goat anti-human IgM Fc µ-chain conjugated to horseradish peroxidase (HRP) (Jackson ImmunoResearch, West Grove, PA, USA) diluted in blocking buffer at 1:5000 and then incubated for 1 h and 30 min at RT with a secondary antibody goat anti-mouse IgG 2a heavy chain conjugated to horseradish peroxidase (Abcam, Cambridge, UK) diluted in blocking buffer at 1:5000. The goat anti-human IgM Fc µ-chain antibody recognized the IgM Fc portion of EpCAM–IgM Fc, whereas the anti-human EpCAM antibody detected EpCAM. Protein bands were visualized by exposing the membrane to X-ray film (Fuji, Tokyo, Japan) using a chemiluminescence substrate (Pierce). Non-transgenic plants and tobacco-derived human EpCAM–IgM Fc (50 ng) [52] were used as negative and positive controls, respectively.

### 4.6. Cross-Fertilization

After T_0_ transgenic plant validation by PCR and Western blot analysis, the transgenic plantlets (T_0_) with well-developed roots were transplanted into 10 cm pots containing soil and transferred to a glasshouse at the National Institute of Horticultural and Herbal Science (NIHHS), Wanju, Korea from 2015 to 2017 (Figure 1B). The plants were grown under normal daylight conditions with a set temperature of 22 °C and 60–70% relative humidity (RH). The T_0_ plants were grown in 2015 and selfed to produce seeds (T_1_). In 2016, the T_1_ seeds were sown and the plants were grown for 4–6 weeks with vernalization treatment (Figure 1C). The T_1_ plants containing the EpCAM–IgM Fc genes were crossed with the T_1_ plants with J-chain K during the blooming stage for the generation of the F_1_ transgenic lines in 2017. The crossing resulted in the generation of the F_1_ transgenic line (Figure 1D).

### 4.7. Enzyme-Linked Immunosorbent Assay (ELISA)

The Maxisorp 96-well immuno plates (Sigma-Aldrich, St. Louis, MO, USA) were coated and incubated overnight at 4 ℃ with 10 ng per well of purified tobacco plant-derived EpCAM–IgG Fc (EpCAM–IgG Fc^T^) [44], tobacco plant-derived EpCAM–IgM Fc (EpCAM–IgM Fc^T^) × J-Chain K^T^ [48], and Chinese cabbage-derived EpCAM–IgM Fc (EpCAM–IgM Fc^C^) × J-Chain K^C^ proteins diluted in 0.05 M carbonate/bicarbonate buffer (Sigma-Aldrich, St. Louis, MO, USA). After overnight incubation, the plates were washed with 1X PBS plus 0.5% (*v/v*) Tween 20 (1X PBS-T) four times and blocked with 5% skim milk (Sigma-Aldrich, St. Louis, MO, USA) in 1X PBS-T for 1 h at RT. Then, the plates were treated with rabbit anti-human IgG Fcγ antibody conjugated to HRP (Jackson Immunolab, West Grove, PA, USA) or goat anti-human IgM Fc µ-chain antibody conjugated to HRP (Abcam, Cambridge, UK) diluted 1:5000 in 1X PBS-T or 1X PBS and incubated for 2 h at RT. After washing four times with 1X PBS-T, the plates were detected using 3,3′,5,5′-Tetramethylbenzidine (TMB) substrate and TMB stop solution (SeraCare, Milford, CT, USA). The plates were read using an Epoch Microplate Spectrophotometer (BioTek, Winooski, VT, USA) at 450 nm.

### 4.8. Statistical Analysis

Statistical analysis consisted of using Student’s *t*-test to determine the differences in absorbance of each group (EpCAM–IgG Fc^T^, EpCAM–IgM Fc^T^ × J-Chain K^T^, and EpCAM–IgM Fc^C^ × J-Chain K^C^) using Microsoft Excel software (Microsoft Office Excel; Microsoft Corporation, Redmond, WA, USA). The difference between each group (EpCAM–IgG Fc^T^, EpCAM–IgM Fc^T^ × J-Chain K^T^, and EpCAM–IgM Fc^C^ × J-Chain K^C^) was compared for statistical significance at 0.05 and 0.01 probabilities (* *p* < 0.05, ** *p* < 0.01).

## Abbreviations

ELISA	Enzyme-linked immunosorbent assay
ER	Endoplasmic reticulum
EpCAM	Epithelial cell adhesion molecule
Fc	Fragment crystallizable
IgM	Immunoglobulin M
J-chain	Joining-chain
HRP	Horseradish peroxidase
MS	Murashige and skoog
PBS	Phosphate-buffered saline
PCR	Polymerase chain reaction
TAA	Tumor-associated antigen
